# Supplementary material for: Microfluidics with fluid walls
Source: Nat Commun. 2017 Oct 10;8:816. doi: 10.1038/s41467-017-00846-4 (PMC5635017; doi:10.1038/s41467-017-00846-4)
Supplement: Supplementary file 1 — Supplementary Information [file 41467_2017_846_MOESM1_ESM.pdf]

SUPPLEMENTARY INFORMATION

SUPPLEMENTARY FIGURES

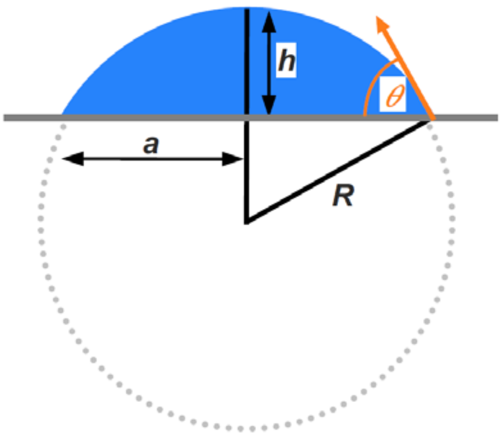

Supplementary Figure 1. An aqueous drop (blue area) sitting on a flat substrate.

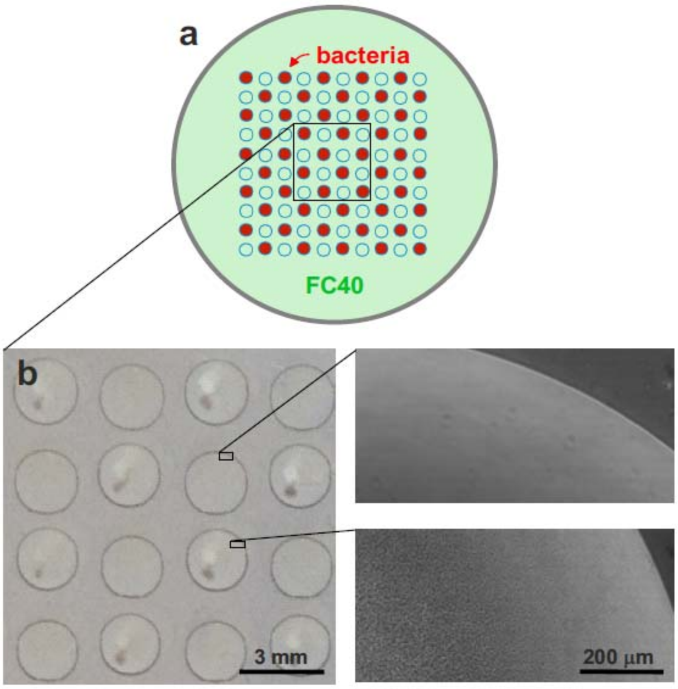

Supplementary Figure 2. Using FC40 to isolate drops.
(**a**) Overview. A 10x10 array of 1-µl drops of growth medium was printed on a 6-cm dish, *E. coli* added to every second drop, and the dish overlaid with FC40; after incubation (1 d at 37°C, and then 1 d at ~20°C), the dish was photographed.
(**b**) Image of some drops. Bacteria grow only in inoculated drops as the rest remain sterile. Flow within a drop causes some bacteria to aggregate, giving cloudy and dark regions. Every second drop contains bacteria-free medium, and these remain visibly clear (even after 10 days sitting on a laboratory bench). Insets: phase-contrast magnifications of selected areas (granularity indicates the presence of bacteria).

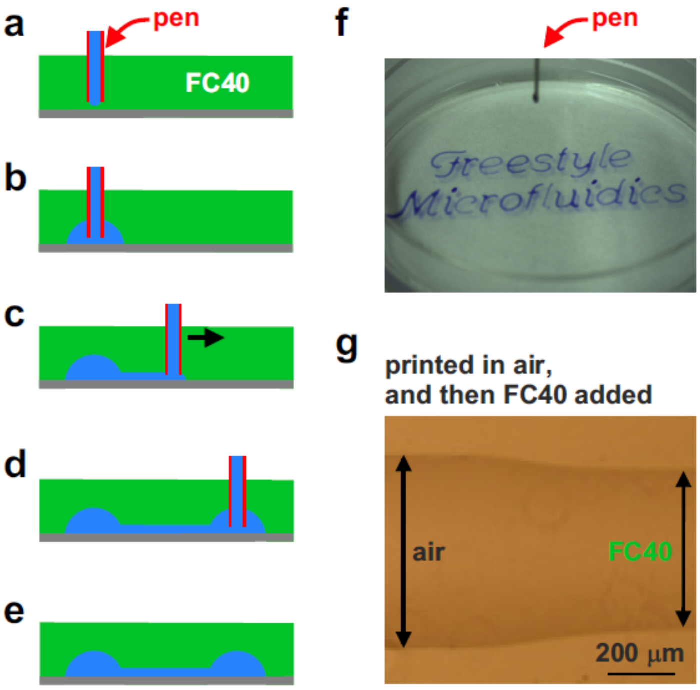

Supplementary Figure 3. Creating simple circuits under FC40.
(**a-e**) Principle. (a) A pen filled with water is brought close to a substrate covered with FC40, (b) water is ejected to deposit a drop, and (c) the pen is moved laterally to create a conduit; (d) after stopping the pen, a second drop is deposited, and (e) the pen is withdrawn.
(**f**) Example circuit printed under FC40 in a 6-cm dish using tissue-culture medium containing blue dye and a hollow stainless-steel needle (outside diameter 0.5 mm).
(**g**) Effects of FC40 on conduit width. A straight conduit was printed in air from left to right, and then FC40 was added as printing continued; finally, a phase-contrast image was collected of the segment of the conduit that was being printed as FC40 flowed over it. Conduit width falls from left to right. This is because the pen printed in air on the left, and under FC40 on the right (which increases the wetting angle).

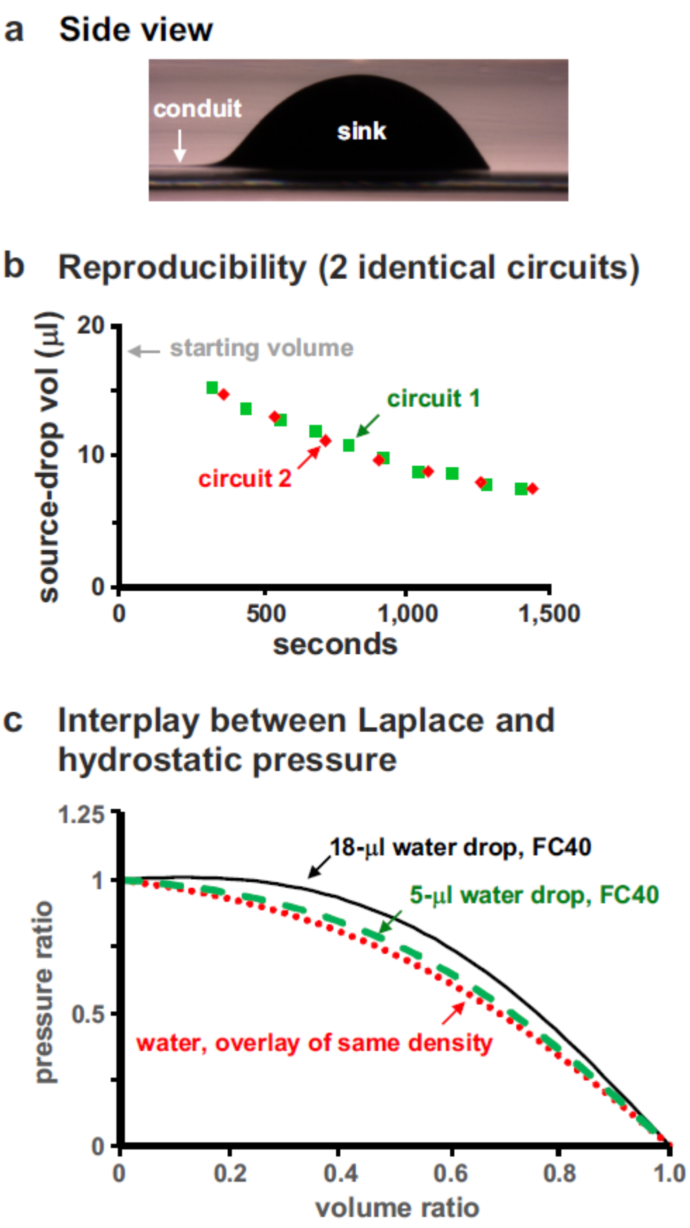

Supplementary Figure 4. Characterizing flows in circuits. Circuits are like the ones in Figure 2d (18 µl source drops connected to 20 µl sinks through 11 mm conduits).

a. Side view of part of a conduit and sink drop.

b. Reproducibility of flow between source and sink drops. Two identical circuits were made with a conduit that had a width of 1,100 µm (3 mm FC40 overlay). Time-lapse imaging (side views) show volumes of source drops decrease over time; these volumes were determined using images like the one in (a). Volumes of source drops in the two circuits fall at the same rates.

c. Interplay between Laplace and hydrostatic pressures affects source-drop volume. See Supplementary Note 2.

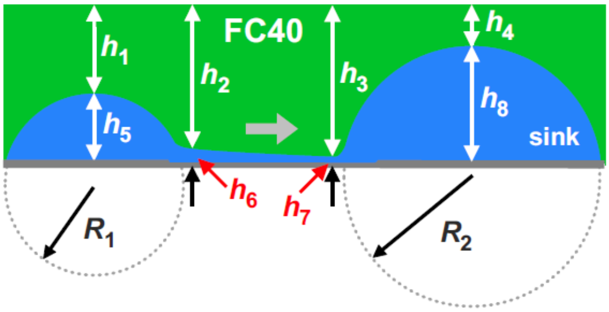

Supplementary Figure 5. Interplay between Laplace and hydrostatic pressures. This Figure amplifies Figure 2e; see also Supplementary Note 1. The left-hand drop has a smaller radius of curvature (and so higher Laplace pressure) than the right-hand one, and is overlaid with a greater height of FC40 (and so has a higher hydrostatic pressure); here, both pressures combine to drive flow to the right (grey arrow).

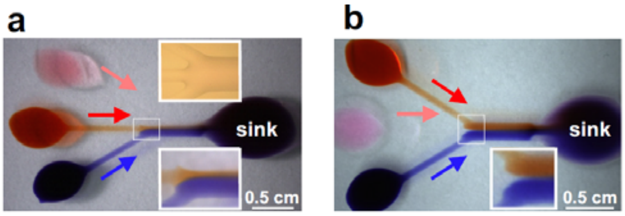

Supplementary Figure 6. Reusing a circuit. Colored arrows illustrate flows driven mainly by differences in Laplace pressure, and insets show how well pinning lines persist.
(**a**) Reproduction of the image shown in **Figure 3c**. This circuit was used for efficient flow-focusing of the central laminar stream after the junction.
(**b**) The same circuit used in (a) was reused here after pipetting red dye into the topmost left-hand drop and medium (pink) into the middle left-hand drop – a process which was repeated until colors were reversed.

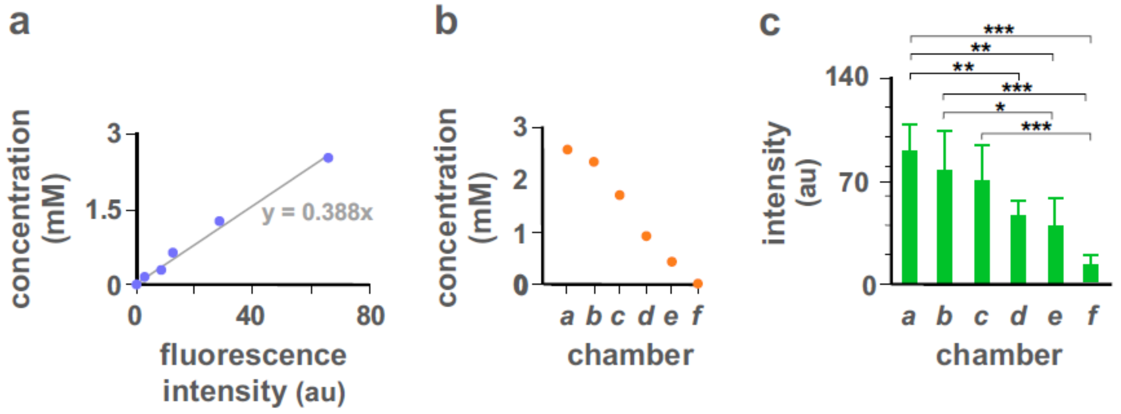

Supplementary Figure 7. HEK cells in an FF circuit respond normally to TNFα. This data supports Figure 4b and c.

(**a**) Characterizing serial dilutions using fluorescein as a surrogate for TNFα. The circuit was printed and operated as described in Methods except that cells were omitted and 9.5 µl 5 mM fluorescein and 9.5 µl water were immediately added to source drops 1 and 2 instead of media + TNFα and media. Then, after 24 h incubation at 37°C, the Petri dish was placed on a fluorescent microscope and fluorescent intensities of chambers measured (au: arbitrary units). To relate fluorescent intensity to fluorescein concentration in a chamber, the same circuit was made but conduits connecting chambers contained breaks (so chamber volumes could not change), and chambers were filled with 3.6 µl containing serial dilutions of fluorescein (starting at 5 mM). After another 24 h incubation, fluorescence intensities were measured using the same settings as before. The linear calibration curve relates fluorescence intensity to molar concentration of fluorescein in each chamber (y = 0.0388x).

(**b**) The concentration of fluorescein in each chamber calculated using the standard curve in (a).

(**c**) The fluorescence intensities given by HEK cells in chambers *a-f* after treatment with TNFα as in Figure 4c. Intensities (in arbitrary units, au, ± SD) were measured as described in Methods (no background was subtracted in *f*) using 3 circuits and cells plated on the same day. The concentrations of TNFα in chambers *a-f* were 5.1, 4.7, 3.4, 1.8, 0.8, and 0 ng/ml; they were calculated using data in (a) and (b). *, **, and ***: the probability that the differences in intensity seen in the pairs of samples indicated arose by chance was <0.05, <0.01, and <0.001, respectively (one-way Anova, Tukey's test).

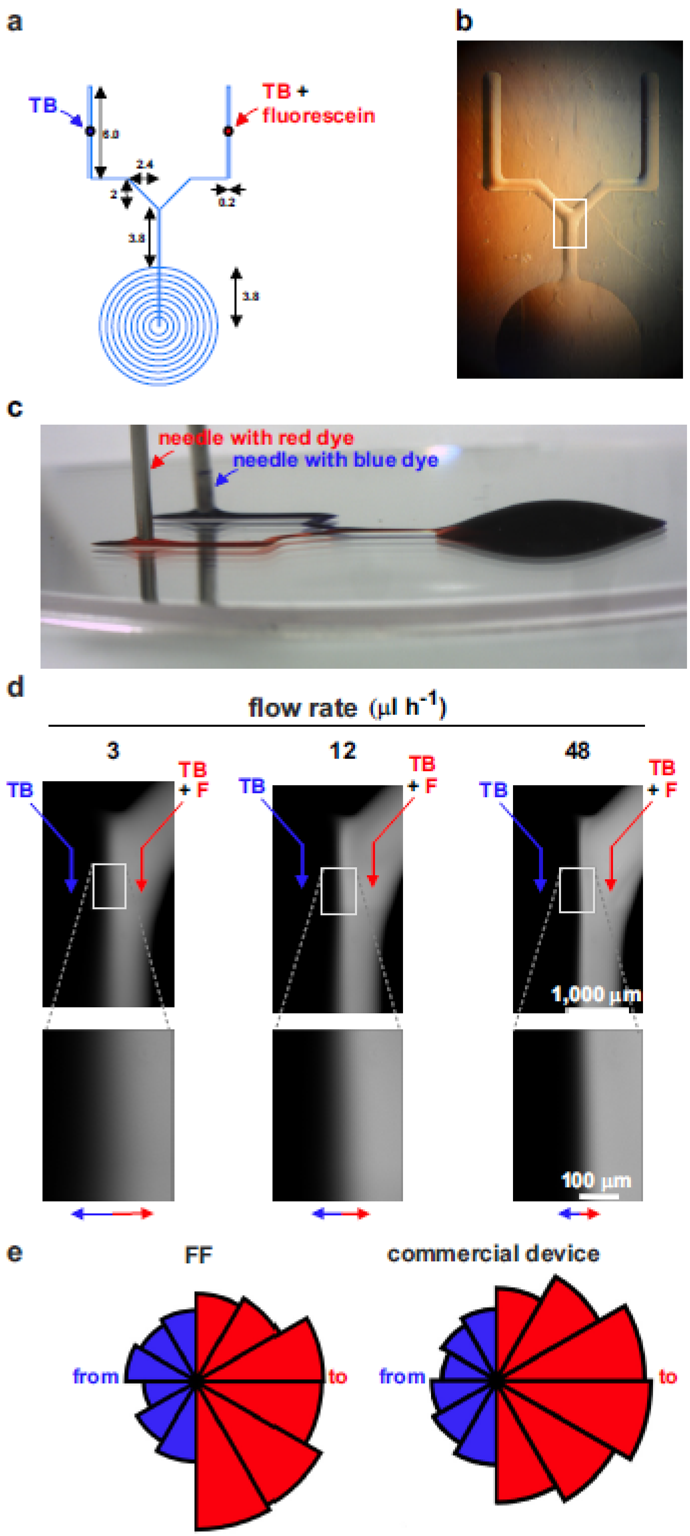

Supplementary Figure 8. Manufacture and operation of the circuit used to analyse bacterial chemotaxis.

(**a**) Plan of the circuit used in Figure 5 (dimensions in mm). Blue lines outline the path taken by a 0.5-mm hollow needle as it traversed (20 mm/sec) above a 40-mm cell-culture dish with a glass bottom ejecting fluid (flow rate of 200 nl/sec) on to the surface. On printing, closely-spaced lines merge to give two input conduits (spacing between center-lines of 9 mm, a central conduit (length 3.8 mm; only a small part of this is imaged), and a flat sink drop (bottom).

(**b**) Bright-field view of the circuit. Rectangle: region imaged in (d).

(**c**) View through the side of the dish filled with FC40 showing the circuit in operation, when red and blue dyes (instead of TB and TB + fluorescein) are being pumped through hollow needles into the circuit.
(**d**) Fluorescence images of the region shown in (b). Prior to imaging, the dish was placed on a confocal microscope, hollow needles inserted into the two input channels (see Fig. 5a), and TB or TB + fluorescein pumped into the circuit (using 2 syringes connected to one pump) at the rate indicated. Insets: magnifications of regions indicated (colored arrows at the bottom point to highest concentrations of TB and DMSO). After the junction, TB and TB + fluorescein flow side by side as laminar streams. At a flow rate of 3 μl/h, there is time for considerable amounts of fluorescein to diffuse to the left to create a shallow gradient; with 48 μl/h, there is less time for dye diffusion, and this creates a steeper concentration gradient.
(**e**) Comparison of bacterial chemotaxis within an FF device with that obtained using a commercially-available PDMS-based device (Bioflux, Fluxion Biosciences). The rose plot obtained with the FF device reproduces that shown in Figure 5d, while that obtained with the commercial device is derived from data collected by Oliveira *et al*.[29]. Both plots were compiled using trajectories collected in two separate fields of view over the first 6 h of the experiment. Each sector denotes the probability density function of the angle from each trajectory's origin to its final position, with red/blue bins denoting movement towards/away from DMSO. The chemotactic response in the two devices is similar.

SUPPLEMENTARY NOTES

Supplementary Note 1: Drop dimensions, contact angles, Laplace and hydrostatic pressures

Drop dimensions, and flow through FF circuits (often driven by differences in Laplace and hydrostatic pressures) are now discussed with reference to **Supplementary Figure 1**, where θ is the contact angle.

The Laplace pressure across the drop interface depends on *R*; *R* can be calculated using Pythagoras' theorem.

$$(R-h)^2 + a^2 = R^2 \Rightarrow R = \frac{h^2+a^2}{2h} \qquad 1$$

h and *r* can be determined using trigonometry:

$$h = R(1-\cos\theta) \qquad 2$$

$$a = R\sin\theta \qquad 3$$

$$h = \frac{a}{\sin\theta}(1-\cos\theta) \qquad 4$$

The volume, V_{cap}, and surface area, A_{cap_surf}, of a cap of a sphere (drop) are:

$$V_{cap} = \frac{\pi h}{6}(3a^2+h^2) \xrightarrow[a^2=2Rh-h^2]{} \frac{\pi h^2}{3}(3R-h) \qquad 5$$

$$A_{cap\_surf} = 2\pi Rh \xrightarrow[Rh=\frac{h^2+a^2}{2}]{} \pi(a^2+h^2) = \frac{2\pi h^2}{1-\cos\theta} = \frac{2\pi a^2(1-\cos\theta)}{\sin^2\theta} \qquad 6$$

V_{cap} can also be expressed in terms of contact angle (θ), drop height (*h*), and radius of wetted footprint (*a*):

$$V_{cap} = \frac{\pi(2-3\cos\theta+\cos^3\theta)}{3(1-\cos\theta)^3}h^3 \xrightarrow[h=\frac{a}{\sin\theta}(1-\cos\theta)]{} \frac{\pi(2-3\cos\theta+\cos^3\theta)}{3}\frac{a^3}{\sin^3\theta} \qquad 7$$

To determine the surface area of the drop wetting the culture dish (needed, for example, when estimating cell-seeding numbers) using the volume of drop deposited, the wetted radius (*a*) and area of the dish covered by the drop (A_{wetted}) are:

$$a = \left[\frac{3V_{cap}\sin^3\theta}{\pi(2-3\cos\theta+cos^3\theta)}\right]^{\frac{1}{3}} \qquad 8$$

$$A_{wetted} = \pi a^2 = \pi\left[\frac{3V_{cap}\sin^3\theta}{\pi(2-3\cos\theta+\cos^3\theta)}\right]^{\frac{2}{3}} \qquad 9$$

The surface area in contact with air or FC 40, A_{cap_surf}, is:

$$A_{cap\_surf} = \frac{2\left[\frac{3V_{cap}\sin^3\theta}{\pi(2-3\cos\theta+\cos^3\theta)}\right]^{\frac{2}{3}}(1-\cos\theta)}{\sin^2\theta} \qquad 10$$

For drops in air with a contact angle of 50° (with volume in microliters):

$$a = 1.08V_{cap}^{\frac{1}{3}}\ mm;\ A_{wetted} = 3.7V_{cap}^{\frac{2}{3}}\ mm^2; h = 0.5V_{cap}^{\frac{1}{3}}\ mm \quad 11$$

When under FC40 with a contact angle of 70°:

$$a = 0.92V_{cap}^{\frac{1}{3}}\ mm;\ A_{wetted} = 2.7V_{cap}^{\frac{2}{3}}\ mm^2;\ h = 0.64V_{cap}^{\frac{1}{3}}\ mm \quad 12$$

Now consider a conduit. Its cross-sectional area, $A_{cross\ section}$, can be estimated from the blue area of **Supplementary Figure 1**:

$$A_{cross\ section} = \underbrace{\pi R^2\left(\frac{2\theta}{360}\right)}_{area\ of\ sector} - \underbrace{a(R-h)}_{area\ of\ triangle} = \frac{1}{2}R^2\left(\frac{\theta\pi}{90} - \sin 2\theta\right) = \frac{a^2}{2\sin^2\theta}\left(\frac{\theta\pi}{90} - \sin 2\theta\right) \quad 13$$

Similarly, the arc length of the interface, $L_{segment}$, is:

$$L_{segment} = \frac{\theta\pi R}{90} = \frac{\theta\pi a}{90\sin\theta} \quad 14$$

If two drops are connected by a conduit, the rate of fluid transfer between drops is proportional to the pressure difference between them (which is due to differences in Laplace and hydrostatic pressures) and the geometry of the conduit. The Laplace pressure (ΔP_L) for a sphere, or cap of a sphere, is given by

$$\Delta P_L = \frac{2\gamma}{R} \quad 15$$

where γ, R, and P are the interfacial tension, radius of curvature, and pressure. The pressure difference between drops due to hydrostatic pressure is given by:

$$\Delta P_{hydrostatic} = \Delta\rho g \Delta h \quad 16$$

where $\Delta\rho$ is the density difference between the drop and surrounding fluid, and Δh the height difference between drops. The Laplace and hydrostatic pressures are summed to provide the net pressure difference driving flow. This approach can be extended to any number of drops to determine relative pressures.

Now consider the circuit in **Supplementary Figure 5**.

$$\Delta P_{interface\_drop} = \frac{2\gamma}{R_{drop}} \quad 17$$

Then, the smaller drop (with smaller radius of curvature) has a larger pressure difference across its interface than the larger one. As drops are overlaid with different heights (h_1, h_4) of denser FC40 (density, ρ), the pressure at the base of a drop results from both hydrostatic and Laplace pressures. The resultant pressure difference between the two drops at the base is

$$\Delta P_{drops} = \rho_{FC40}g(h_1 - h_4) + \rho_{water}g(h_5 - h_8) + \frac{2\gamma}{R_1} - \frac{2\gamma}{R_2} \quad 18$$

Next, consider conduit geometry. The cross section at any point along the conduit is approximated by a segment of a circle; as one radius of curvature is negligible, the Laplace pressure across the interface is

$$\Delta P_{conduit} = \frac{\gamma}{R_{conduit}} \qquad 19$$

We estimate the radius of curvature ($R_{conduit}$) of the inlet (where height is h_6; **Supplementary Fig. 5**) by assuming pressures at the base of a drop and conduit near the inlet are equal. The pressure drop across the conduit interface, assuming conduit height is small relative to drop height, is given by

$$\frac{\gamma}{R_{conduit}} = P_{base(drop)} - \rho_{(FC)} g(h_5 + h_1) = \frac{2\gamma}{R_1} - \Delta\rho_{(FC/water)} g h_5 \qquad 20$$

Once the radius of curvature of the conduit is known, the geometry of the cross section may be calculated (results of example calculations are provided in **Table 2**).

Supplementary Note 2: Detail for Supplementary Figure 4c.

The approximately linear relationship between source drop volume and time in Figure 2d requires an approximately constant flow rate through the conduit, and hence constant pressure in the source drop (assuming the pressure change in the larger sink drop is negligible). However, as the initial drop volume reduces, with a fixed pinning line, the Laplace pressure across the interface also reduces due to the increase in curvature. When the overlaid fluid is denser than the drop fluid, this reduction in Laplace pressure is countered by an increase in hydrostatic pressure. Supplementary Figure 4c illustrates this effect, and more detail is provided here. The pressure and volume ratios are defined as

$$Pressure\ ratio = \frac{P_{base\ of\ drop} - P_{base(FC40)}}{P_{initially\ at\ base\ of\ drop}} \qquad 21$$

$$Volume\ ratio = \frac{V_{drop\ initially} - V_{drop}}{V_{drop\ initially}} \qquad 22$$

In Supplementary Figure 4c, the red dashed line indicates the change in pressure ratio when the overlaid and drop fluids have the same density, and then only the Laplace-pressure change causes a change in pressure ratio. The dashed and solid black lines represent the pressure ratio when the source drops are either 5 or 18 µl and overlaid with denser FC40. For the 18-µl drop, the pressure ratio is essentially constant (< 1% variation) until the volume reduces by ~25%. However, for the smaller drop, the pressure ratio reduces by ~8% at a volume reduction of ~25%. Hence, larger drops maintain a pressure ratio, and hence a steady flow rate, for a greater percentage of their original volume, when overlaid with a denser fluid. When the overlay fluid is equal to or less dense than the drop fluid, the pressure ratio – and hence flow rate – can only decrease as liquid flows from the source drop. A similar consideration would apply to the sink drop in the case where its volume change was not considered negligible.

Supplementary Note 3: software

This provides an example of the G-code program used to print the circuit in **Figure 4b**.

```
% use 0.5 mm external diameter dispensing tip – Created 14/1/2017 Prof. Walsh –
edmond.walsh@eng.ox.ac.uk

M30

G98 L1
G90
G00 x0 y0 z-15
G01 y0 F20
G01 y-1.7
G01 z0
G91
G01 y1.7
G02 x0 y0 i0 j-1.8
G01 y-0.5
G02 x0 y0 i0 j-1.3
G01 y-0.5
G02 x0 y0 i0 j-0.8
G01 y-1.3
G90
G01 x0.25
G01 y18
G01 x-0.25
G01 y0
G01 x0
G01 y18
G01 z-5
G00 y24.3
G00 x13.5
G01 z0
G91
G01 y-1.8
G02 x0 y0 i0 j1.8
G01 y0.5
G02 x0 y0 i0 j1.3
G01 y0.5
G02 x0 y0 i0 j0.8
G01 y1.4
G90
G01 x13.25
G01 y4.5
G01 x13.75
G01 y22.5
G01 x13.5
G01 x13.5
G01 y18.1
G01 x8.55
G01 y18
```

```
G91
G02 x0 y0 i-1.8
G01 x-0.5
G02 x0 y0 i-1.3
G01 x-0.5
G02 x0 y0 i-0.8
G01 x-0.5
G90
G01 y18.1
G01 x0
G01 y17.9
G01 x13.5
G91
G01 x4.95
G01 y0.1
G02 x0 y0 i1.8
G01 x0.5
G02 x0 y0 i1.3
G01 x0.5
G02 x0 y0 i0.8
G01 x1
G01 y0.1
G90
G01 x13.5
G01 y13.6
G01 x8.55
G01 y13.5
G91
G02 x0 y0 i-1.8
G01 x-0.5
G02 x0 y0 i-1.3
G01 x-0.5
G02 x0 y0 i-0.8
G01 x-0.5
G90
G01 y13.6
G01 x0
G01 y13.4
G01 x13.5
G01 y9.1
G01 x8.55
G01 y9.0
G91
G02 x0 y0 i-1.8
G01 x-0.5
G02 x0 y0 i-1.3
G01 x-0.5
G02 x0 y0 i-0.8
G01 x-0.5
G90
G01 y9.1
```

```
G01 x0
G01 y8.9
G01 x13.5
G01 y4.6
G01 x8.55
G01 y4.5
G91
G02 x0 y0 i-1.8
G01 x-0.5
G02 x0 y0 i-1.3
G01 x-0.5
G02 x0 y0 i-0.8
G01 x-0.5
G90
G01 y4.6
G01 x-4.95
G01 y4.5
G91
G02 x0 y0 i-1.8
G01 x-0.5
G02 x0 y0 i-1.3
G01 x-0.5
G02 x0 y0 i-0.8
G01 x-1
G90
G01 y4.4
G01 x13.7
G00 z-20
G00 y50 x50
M99 (End subprogramme)
```
