# Supplementary material for: Microfluidics with fluid walls
Source: Nat Commun. 2017 Oct 10;8:816. doi: 10.1038/s41467-017-00846-4 (PMC5635017; doi:10.1038/s41467-017-00846-4)
Supplement: Supplementary file 3 — Description of Additional Supplementary Files [file 41467_2017_846_MOESM3_ESM.pdf]

Description of Additional Supplementary Files

File Name: Supplementary Movie 1
Description: Printing an FF circuit in air. The movie runs in real time. The pen prints tissue-culture medium containing blue dye on to the surface of a 6-cm polystyrene tissue-culture dish. Figure 1d illustrates the last frame of this movie.

File Name: Supplementary Movie 2
Description: An FF circuit overlaid with FC40 can survive violent agitation. The movie runs in real time. A circuit containing blue dye was printed on a 6-cm Petri dish, overlaid with FC40, placed on a shaker, and the lid of the Petri dish removed. The movie begins when the shaker was started; as the speed of shaking increases, waves form in the FC40 and increase in height, and finally FC40 spills out of the dish.

File Name: Supplementary Movie 3
Description: Using Laplace pressure to drive flow through FF circuits in seconds. This time-lapse movie is speeded up 20x or 120x as indicated. Figure 2b illustrates a frame from this movie. Six identical circuits were printed on a 6-cm tissue-culture dish, and overlaid with 5 ml FC40. A plan of the circuit was now placed under the circuit (dimensions in cm), and then removed. The movie begins when 20 µl tissue-culture media was added to each sink drop on the right (only the last addition is seen). Next, 10, 8, 6, 4, and 2 µl red dye are pipetted successively into left-hand drops (bottom to top). Differences in Laplace pressure drive dye from left to right. Although dye was added first to the bottom left-hand drop, it is nevertheless dye added last that reaches a sink first.

File Name: Supplementary Movie 4
Description: Flow driven largely by Laplace pressure. This time-lapse movie is speeded up 360x. The circuit had the plan illustrated in Figure 2a (left- and right-hand drops contained 10 and 20 µl, respectively), and was overlaid with FC40. The movie (side view) begins just before 10 µl blue dye was manually pipetted into the left-hand drop; then, pressure differences drive dye to the right. The footprint remains the same as the left-hand drop shrinks, and the right-hand one grows.

File Name: Supplementary Movie 5
Description: A mixing circuit. This time-lapse movie is speeded up 60x, and initially there was no FC40 overlay. A frame from this movie is illustrated in Figure 3a. The movie begins as colored dyes are manually pipetted into peripheral drops, and then medium into the central one. Differences in pressure drive dyes towards the central drop where they mix. Towards the end of the movie, the circuit is overlaid with FC40.

File Name: Supplementary Movie 6
Description: A splitting circuit that uses gravity to drive flow. This time-lapse movie is speeded up 5x, and there is no FC40 overlay. The circuit was printed on a horizontal 6-cm Petri dish in air, blue dye was pipetted into the drop at the top, and the dish positioned vertically; the movie begins as gravity is driving blue dye into the 8 drops at the bottom

File Name: Supplementary Movie 7

Description: A trident used for flow-focusing. This time-lapse movie is speeded up 10x. This movie shows the operation of a circuit like the one described in Supplementary Figure 6 which reproduces a frame from this movie. Flow was initiated by pipetting 10 µl medium (pink) or red and blue dyes into left-hand drops, before 30 µl was added to the sink

File Name: Supplementary Movie 8
Description: HEKs grow normally in a drop of medium overlaid with FC40. Frames from this movie are shown in Figure 4a. About 600 HEKs were plated as a 4-µl drop on a 6-cm dish, the drop overlaid with FC40, and cells grown for 24 h in a conventional CO2 incubator. The dish was now mounted on a microscope in an atmosphere of CO2, and a time-lapse movie made (phase-contrast images were collected every 10 min for 48 h, and the video plays at 7 fps).

File Name: Supplementary Movie 9
Description: Making the FF circuit in Figure 5a. This movie runs in real time. The circuit is printed using DMEM + 10% FCS in air on a 4-cm tissue-culture dish with a glass bottom (tip speed 20 mm/s, flow rate of 200 nl/s). Prior to use it is overlaid with 6 ml of FC40.

File Name: Supplementary Movie 10
Description: Air bubbles are automatically rejected by an FF circuit. The movie runs in real time. The circuit is like that in Figure 5a. An external syringe pump drives red and blue dyes through hollow needles (0.5 mm) at 100 µl/h into the circuit (FC40 overlay). Small and large air bubbles were deliberately introduced into tubes feeding blue and red dyes. During the first half of the movie, these bubbles are automatically rejected by the system; buoyancy differences force them to pinch off from the aqueous circuit and rise to the surface of the FC40, where they are lost to the atmosphere. During the second half of the movie, the circuit operates normally.

File Name: Supplementary Movie 11
Description: P. aeruginosa performing chemotaxis. The time-lapse movie over hours of a biofilm growing in a dish on an inverted microscope. A syringe pump drives flow through the circuit as in Figure 5c-e to generate a steady DMSO gradient by diffusion. The surface-attached bacteria preferentially bias pili-based twitching to move up the gradient until the surface becomes crowded with cells, which ultimately slows movement. Here, the gradient was visualized with Chicago Sky Blue 6B dye, which was imaged at the same time as cells using bright-field microscopy. Background subtraction (performed in Fiji) allowed us to isolate signal from the dye, which was post-processed to appear red.

File Name: Supplementary Movie 12
Description: Overlaying FC40 has no effect on the footprint of an FF circuit. The timelapse movie is speeded up 2x initially, and 32x at the end. A Y-shaped circuit connected to a flat drop is printed, and FC40 manually pipetted twice into the dish (so the circuit can no longer be seen by eye). However, the circuit is revealed by injecting a red dye followed by a blue dye into the arms (dyes flow to the sink). The footprint remains unchanged during the whole process.
